# Best practices for interviewing applicants for medical school admissions: a systematic review

**DOI:** 10.1007/s40037-022-00726-8

**Published:** 2022-09-22

**Authors:** John C. Lin, Anagha Lokhande, Curtis E. Margo, Paul B. Greenberg

**Affiliations:** 1 Program in Biology, Brown University, Providence, RI USA; 2 Division of Ophthalmology, Alpert Medical School, Brown University, Providence, RI USA; 3 Section of Ophthalmology, Providence VA Medical Center, Providence, RI USA; 4 Department of Ophthalmology, Morsani College of Medicine, University of South Florida, Tampa, FL USA; 5 Office of Academic Affiliations, US Department of Veterans Affairs, Washington, DC USA

**Keywords:** Interview, Communication skills, Admission, Medical schools

## Abstract

**Introduction:**

Interviews are commonly used to select applicants for medical school, residency, and fellowship. However, interview techniques vary in acceptability, feasibility, reliability, and validity. This systematic review investigated the effectiveness of different interview methods in selecting the best qualified applicants for admission to medical school and developed a logic model to implement best practices for interviewing.

**Methods:**

Five electronic literature databases were searched for comparative studies related to interviewing in medical schools from inception through February 1, 2021. Inclusion criteria included publications in English that compared different methods of conducting a selection interview in medical schools with a controlled trial design. General study characteristics, measurement methodologies, and outcomes were reviewed. Quality appraisal was performed using the Medical Education Research Study Quality Instrument (MERSQI) and the Oxford Risk of Bias Scale. Based on these findings, a logic model was constructed using content analysis.

**Results:**

Thirteen studies were included. The multiple mini-interview (MMI) was reliable, unbiased, and predicted clinical and academic performance; the virtual MMI increased reliability and lowered costs. For unstructured interviews, blinding interviewers to academic scores reduced bias towards higher scorers; student and faculty interviewers rated applicants similarly. Applicants preferred structured over unstructured interviews. Study quality was above average per the MERSQI, risk of bias was high per the Oxford scale, and between-study heterogeneity was substantial.

**Discussion:**

There were few high-quality studies on interviewing applicants for admission to medical school; the MMI appears to offer a reliable method of interviewing. A logic model can provide a conceptual framework for conducting evidence-based admissions interviews.

**Supplementary Information:**

The online version of this article (10.1007/s40037-022-00726-8) contains supplementary material, which is available to authorized users.

**Disclaimer** The views expressed here are those of the authors and do not necessarily reflect the position or policy of the US Department of Veterans Affairs or the US government.

## Introduction

Interviews are an important process in medical education [1]. In the United States (US), there are 94,243 students in medical school [2], most of whom go through an interview as part of the selection process. The selection interview allows admissions officers to verify and supplement other parts of an applicant’s portfolio (grades, standardized exam scores, essays) by gathering information about their personal qualities and capabilities, including proficiency in oral communication, social skills, and motivations that may be predictive of academic success in medical school and future clinical performance. Additionally, interviews are a way of exhibiting institutional environments and encouraging candidates to matriculate at schools and training programs.

Interviews in medical education can be problematic, however. They are less reliable and predictive of medical trainee academic and clinical performance than grade point averages (GPA) and national examination scores [3–8]. Moreover, many aspects of the interviewing process may discriminate against applicants based on certain characteristics such as race, gender, and other demographic factors [9]. In addition, in-person interviews are time consuming and can pose a significant financial burden for prospective trainees. These counterbalancing features underscore the importance of determining the most effective ways of conducting fair and reliable interviews that can identify the best qualified applicants for training in medicine.

Evaluating evidence-based approaches to interviewing will help medical education programs reduce bias and admit more qualified applicants. However, prior systematic reviews on best approaches to interviewing in medical education have largely focused on the reliability and validity of the multiple mini-interview (MMI), which is used to evaluate different aspects of an applicant’s character with short interview stations [10, 11]. The primary purpose of this systematic review was to compare the effectiveness of different interview methods in selecting the best qualified applicants for medical school. The secondary purpose was to use the findings of the systematic review to devise a logic model to guide the implementation of evidence-based interview practices [12, 13].

## Methods

A health sciences librarian was consulted to formulate search strategies in accordance with Preferred Reporting Items and Meta-Analyses (PRISMA) guidelines. A literature search was performed for randomized controlled trials (RCTs) and comparative observational studies using CINAHL, Embase, ERIC, PsycINFO, and PubMed from inception to February 1, 2021. (See Electronic Supplementary Material 1 [ESM 1] for searches).

Two investigators (JL, AL) independently assessed each study in Covidence (https://www.covidence.org/) for full-text review by screening their titles and abstracts. After conflicts were resolved, JL and AL independently conducted a full-text analysis for eligibility. JL and AL independently extracted the following information from the articles: publication year, country, study design, demographic information, sample size, setting, interview techniques, and primary outcomes. Study countries were categorized based on their Global Burden of Disease (GBD) super-region. All disagreements were resolved by the senior investigators (CEM, PG). To identify additional papers for consideration, we used forward reference searching on Google Scholar to find articles that cited relevant literature reviews [10, 11, 14–17].

Our inclusion criteria included studies that compare different methods of conducting a selection interview in medical schools using a controlled trial design, including RCTs and comparative observational studies. A comparative observational study is defined as a study that tests different interview techniques with two or more distinct comparison groups with minimal differences, such as a parallel group study [18].

Our exclusion criteria included: (1) non-English publications, (2) publications that report on the selection interview in non-medical school settings, (3) observational studies, (4) studies that test different interview techniques using the same sample, and (5) reviews, editorials, case studies, and reports. Crossover studies were excluded as their designs may be inherently prone to a higher risk of bias due to carry-over effects [19, 20]. For example, if applicants were interviewed twice using different formats by the same interviewer, their first performance will affect their score for the second interview; additionally, even if the interviewer was different, the applicant may have gained additional experience and familiarity with interview questions from the first interview.

JL and AL independently evaluated the quality and risk of bias of the studies using the Medical Education Research Study Quality Instrument (MERSQI) and the Oxford Risk of Bias Scale, respectively. MERSQI scores range from 0 to 18 based on study design (0–3), sampling (0–3), type of data (0–3), validity evidence (0–3), data analysis (0–3), and outcome reporting (0–3) [21]. The Oxford scale, also known as the Jadad scale, is the most frequently used metric for risk of bias in the medical literature and provides scores from 0 to 5 based on randomization (0–2), blinding (0–2), and attrition (0–1) [22]. Disagreements were resolved by the senior authors.

Logic models are designed to visually map the relationship between interventions and their short-, intermediate-, and long-term outcomes [12, 13]. A logic model was developed by synthesizing the findings of the included studies using content analysis. Categories of interview methods were created to classify interview content and planning for the logic model. Next, methodologies of included studies were reviewed, approaches to developing interview processes were extracted, and activities necessary to conduct interviews were incorporated into the model. Results were listed and divided into targeted short-term outputs for individuals and long-term outcomes for systems based on the principles of logic model development for systematic reviews [13].

## Results

**Fig. 1:**
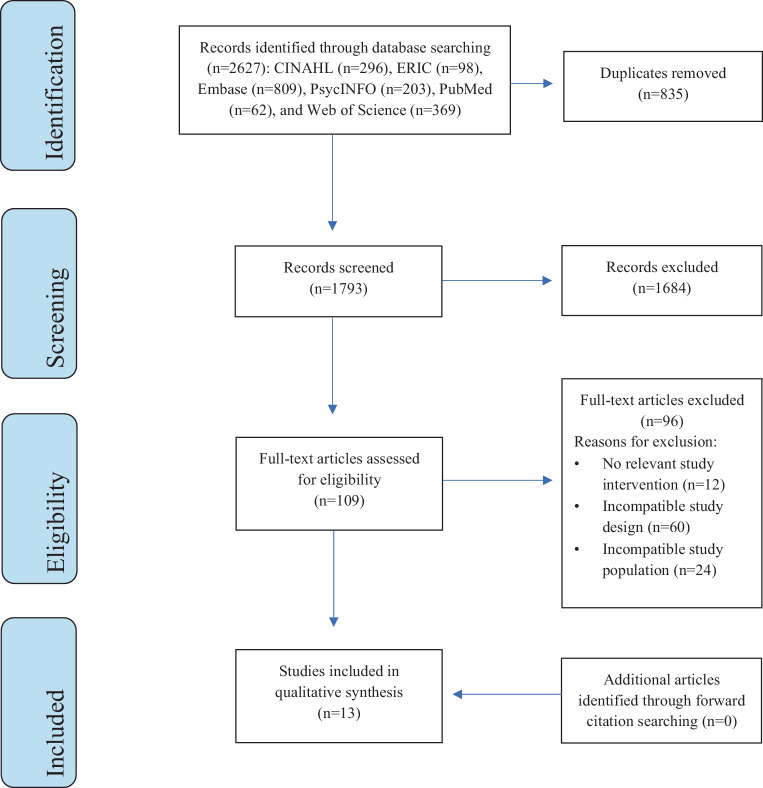
PRISMA flow diagram

After removing duplicates, 1793 potential studies were identified (Fig. 1). After screening titles and abstracts, the full texts for 109 studies were comprehensively reviewed. In total, 96 were excluded due to incompatible study design (60), incompatible study population (24), and incorrect intervention (12). Forward reference searching was performed but did not identify any eligible studies for systematic review. This strategy yielded thirteen studies included in the review [9, 23–33].

### Included studies

Interventions and study populations varied between studies (ESM 2). Nine (69%) studies examined the MMI [24, 25, 27–29, 31–33], and nine (69%) used a comparative observational study design [9, 23, 25, 27–30, 32, 33]. Eleven (85%) studies were conducted in high-income countries [9, 23–31], and ten (77%) were based at public medical schools [9, 23–25, 27, 28, 30, 32, 33].

Five main outcomes were identified: interview bias, reliability, feasibility, acceptability, and validity (ESM 3). All outcomes, including feasibility (costs), were determined independently by authors of included studies and reported in their manuscripts. Included studies had high risks of bias and high quality of research design (see Tab. 1); MERSQI scores ranged between 12.5 and 17 (maximum = 18) and Oxford scale scores ranged from 0–2 (maximum = 5). MERSQI scores above 12.5 and Oxford scale scores above three have been considered the cut-off for high-quality studies in the literature [21, 34, 35].

**Table 1:** Determination of risk of bias and study quality

Study	Risk of bias^a^	Study design^b^	Sampling^b^	Type of data^b^	Validity of evaluation instrument^b^	Data analysis^b^	Outcomes^b^	Total MERSQI score^b^
*Shaw et al. (1995) *[9]	0	2	2	3	3	3	3	16
*Albanese et al. (2003)* [16]	0	2	1.5	1	2	3	3	12.5
*Reiter et al. (2006): Study 1* [24]	2	3	2	3	2	2	3	15
*Reiter et al. (2006): Study 2* [24]	2	3	2	3	2	2	3	15
*Uijtdehaage et al. (2011) *[25]	0	3	2	3	3	3	3	17
*Eddins-Folensbee et al. (2012) *[26]	2	2	2	3	2	3	3	15
*Husbands et al. (2013) *[27]	0	2	2	3	3	3	3	16
*Tiller et al. (2013) *[28]	0	2	2	3	2	3	3	15
*Hissbach et al. (2014) *[29]	0	2	2	3	3	3	3	16
*Gay et al. (2018) *[30]	0	3	2	3	2	3	3	16
*Kim et al. (2018) *[31]	1	2	2	1	2	3	3	13
*Yusoff et al. (2020) *[32]	0	2	2	1	2	3	3	13
*Yusoff et al. (2020) *[33]	0	2	2	1	2	3	3	13

*MERSQI* Medical Education Research Study Quality Instrument

^a^ Based on the Oxford risk of bias tool

^b^ Based on MERSQI

### The Multiple Mini-Interview (MMI)

The MMI demonstrated little bias, good reliability, moderate costs, strong acceptability, and predictive validity for clinical and academic performance, although most findings were based on outcomes from a single study and thus should be interpreted carefully. The MMI produced similar results regardless of whether some students received summaries of interview questions two weeks prior to their interview [24] or the order in which students rotate through the MMI stations [31]. Station order also did not affect perceived difficulty or anxiety among students either [31]. Making an MMI station more challenging did not show bias based on gender or disadvantaged status and improved reliability (from G = 0.58–0.71) [25]. Additionally, four studies found that MMI ratings had good interview reliability [24, 25, 27–29]. Cost savings could be achieved without sacrificing reliability or creating interviewer bias by reducing the number of MMI stations from twelve to nine (from $915 to $495 per student) and switching to a virtual MMI format (from $61,887 to $10,145 total) [28, 29]. The MMI displayed predictive validity for a first year of medical school year written examination and Objective Structured Clinical Examination (OSCE) scores, especially with more traditional interview stations rather than task-based ones [27]. Although the MMI displayed higher convergence with social self-perception than the semi-structured panel interview [32], the MMI did not show predictive validity for stress, anxiety, depression, or burnout [33].

### The unstructured interview

The unstructured interviews studied in the four (31%) remaining studies had several issues, including potential bias, bad reliability, and low acceptability, although all but one of the outcomes were based on the results of a single study. Unstructured interview ratings were significantly influenced by gender, age, GPA, and MCAT scores [9, 30]. Blinding interviewers to GPA and MCAT scores reduced this bias without affecting reliability (from Cronbach’s α = 0.496–0.473) [9]. However, reliability was unacceptable for unstructured interviews [9]. Unstructured interview ratings were similar between panels of faculty members and mixed panels of students and faculty across six years [26]. Adding structure to an interview by developing standard interview questions, escorting students to meetings across campus, and planning interactions with current students improved student perceptions of the interview process, including its perceived usefulness and thoroughness [23].

## Discussion

### Summary of evidence

This systematic review of controlled studies investigating methods of interviewing found that the MMI approach provided impartial, reliable ratings. Conducting the MMI virtually and asking more difficult questions at MMI stations increased its reliability in single studies. In addition, reducing the number of stations or using virtual interviews saved costs without impacting MMI reliability, although these two findings were based on data from two single-institution studies. Using unstructured interviews, blinding interviewers to academic scores reduced bias; in another study, student interviewers had similar levels of bias to faculty members. In anonymous surveys, applicants preferred structured over unstructured interviews.

All the included studies had high risks of bias based on the Oxford scale but were mostly rated as high quality by the MERSQI. A prior systematic review of simulation training in obstetrics and gynecology similarly found that MERSQI scores were overall higher than Oxford scale scores [36], possibly because the Oxford scale puts great weight on whether the method of randomization was appropriate and whether participants were masked (80% of total score) [22], whereas the MERSQI only asks whether randomization occurred (17% of total score) [21]. Randomization is challenging in interviewing studies as applicants may perceive certain types of interviews as more difficult [37], raising concerns about the fairness of admitting students based on separate interview tracks. Masking is also challenging as applicants may discuss interview experiences and schools often conduct interviews on the same day to conserve resources. Additionally, most RCTs in this review did not report on their method of randomization, leading to further deductions on the Oxford scale. Hence, the differences in Oxford and MERSQI ratings may reflect inherent challenges in medical education research [36] and insufficient transparency regarding methodology.

### Limitations of the evidence base

There were several shortcomings in the current evidence base for interviewing in medical education. First, there were few rigorously designed studies investigating interview techniques other than the MMI and unstructured interviews in medical education. All included studies had high risks of bias. Of our thirteen studies, nine focused exclusively on the MMI. Other common interview methods that were not studied included structured interviewing with hypothetical behavioral or experience-based questions. Second, although quality of evidence was above average for medical education, there were only four RCTs. Third, most studies failed to describe randomization or blinding in sufficient detail, increasing the difficulty of quality assessments [38, 39]. Fourth, many studies did not describe the development of interview questions, making their studies difficult to reproduce [40]. Fifth, few studies assessed key outcomes such as the interviewee’s perception of the interview process, the predictive validity of interview scores for future clinical performance in medical school, or applicant yields based on different interview types. Lastly, study heterogeneity impacted comparison of interventions and outcomes. Almost all studies created their own rating scales for interview performance and interviewee perceptions, which made comparison of interventions from different trials unreliable. Some studies trained interviewers, which may have improved their interrater reliability. Trained students interviewed applicants in several studies; faculty members conducted the interviews in other studies.

### Limitations of the review

Our systematic review was restricted to comparative studies and RCTs published in English; nine of the thirteen included studies were conducted in English-speaking countries. Also, the review focused only on interview types, omitting related topics such as the financial cost of attending interviews and interview weighting in the admission process [2, 41]. In addition, as our studies were focused on medical school admissions, our findings may be less generalizable to the graduate medical education (GME) setting. Studies were conducted mostly in high-income countries and public medical schools; variations in medical school applicant populations may reduce generalizability.

### Implications for medical education

Admissions interviews are used in almost all medical schools despite the weak evidence base [1]. Due to the coronavirus disease 2019 (COVID-19) pandemic, interview procedures are being modified to include virtual interviews [42]. As medical schools revise their interview processes, they should ensure that applicants are aware of the intended purpose of the selection interview [3–8]. The classical test theory, which holds that an applicant’s observed interview performance may not accurately reflect their true personal characteristics due to interview day deviations (e.g., bad weather, difficult circumstances, etc.) [43, 44], can be applied to decide on the weighting of interview scores, examination results, and GPA [3]. Therefore, medical schools should consider reducing the weight of the admissions interview given the lack of strong, multi-study evidence indicating its effectiveness.

The interview of choice is the MMI, which limited bias against applicants, was resistant to question leaks, and had higher reliability when transitioned to a virtual format. One reason may be that most MMI studies trained their interviewers. Another may be that MMI interventions integrated several types of interviewing, such as behavioral, situational, and unstructured interviewing [45]. The MMI’s strong prediction of medical school clinical and academic assessments relative to other admission tools (UK Clinical Aptitude Test, Universities and Colleges Admissions Service) suggests that it holds predictive validity as an interviewing method in medical education.

Previous observational studies on MMI have raised questions about potential bias against applicants who do not speak English as their first language [8], lower reliability with fewer MMI stations [46], and preference for extroverted applicants [47]. However, many of these issues have been identified in traditional interview systems as well [14]. Given the scarcity of RCTs and high-quality studies examining medical school admission interviews, more research is necessary.

### Logic model

**Fig. 2:**
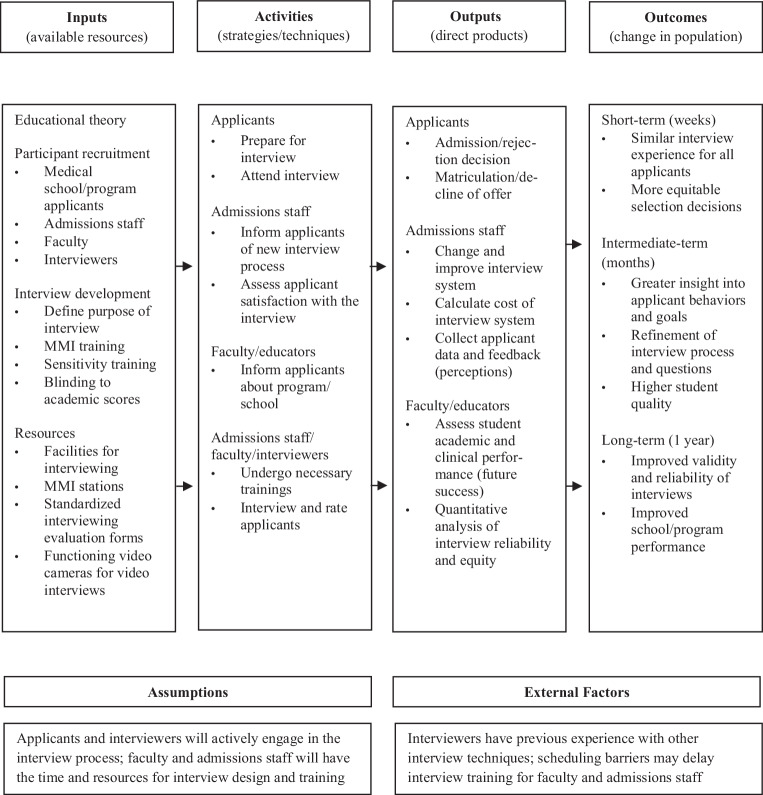
A logic model for how to conduct a medical school admissions interview

We used our findings to construct a logic model (Fig. 2) to develop an admissions interview system and to rigorously evaluate its validity. This conceptual framework highlighted the resources and activities needed to develop and conduct interviews as well as the direct outputs (e.g., admission decisions) and long-term outcomes (e.g., changes in student body).

The key inputs in the model were interviewer time, faculty and staff salaries, interview development, and facilities. The model recommended that personnel and professional psychologists be recruited to help develop structured, behavioral interview questions. It also recommended that the interviewing mode be standardized to mitigate potential differences in interview scoring between virtual, telephone, and face-to-face interviewing [48] and structured to improve organizational attractiveness to applicants [49]. The activities in the logic model included interview meetings, interviewer training, and assessments of interviewee satisfaction with the interview process. Interviewer training helped ensure all interviewees are treated similarly [50]. It was also important to conduct sensitivity and anti-harassment training to mitigate any potential interviewer biases. Ideally, interviewers should be blinded to academic scores so that their ratings purely reflect non-academic traits; blinding also resulted in higher interview ratings for female and minority interviewees [9].

Admissions decisions, matriculation choices, improvements to interview process, and quantitative data were targeted outputs in the model. In the short term, interviewees will receive similar interview experiences and more equitable selection decisions. In the intermediate term, there will be improved understanding of interviewees, a more valid and reliable interviewing process, and a student body that better aligns with the goals of the admissions office. In the long run, schools and programs will improve interview reliability and interviewee perceptions and altered composition of the student body will improve the academic performance of students. The relationship between each approach to interviewing and its outcomes should be assessed to evaluate the effectiveness of an interviewing intervention.

### Conclusions

In summary, despite the widespread use and attendant costs of medical school interviews, there is a paucity of studies that have rigorously examined the role of interviewing in selecting applicants. We recommend further research to address this gap, including examining the equity and predictive value of different selection interview formats and implementing interviewer and outcome assessor training to reduce study heterogeneity. Pending an evidence base with more depth and breadth, we suggest scaling down the relative weight of the interview in the admissions process [7] and using a virtual MMI, which offers an alternative that is safer (i.e., during the COVID-19 pandemic) and reliable while also reducing the financial impact of interviews for applicants, medical schools, and GME programs. We hope our logic model will help educators conduct rigorous admissions interviews for medical school.

## Supplementary Information

ESM 1: Search strategy

ESM 2: Characteristics of included studies and interventions

ESM 3: Intervention and measurement methods of included studies
