# Supplementary material for: Best practices for interviewing applicants for medical school admissions: a systematic review
Source: Perspect Med Educ. 2022 Sep 22;11(5):239–46. doi: 10.1007/s40037-022-00726-8 (PMC9510545; doi:10.1007/s40037-022-00726-8)
Supplement: Supplementary file 1 — ESM 1: Search strategy [file 40037_2022_726_MOESM1_ESM.docx]

**ESM 1: Search strategy**

| **Database** | **Search Query** |
| --- | --- |
| PubMed | ("education, medical"[MeSH] OR "students, medical"[MeSH] OR "medical school*"[tiab] OR "medical student*"[tiab] OR "medical educat*"[tiab]) AND ("interviews as topic"[MeSH] OR "interview*"[tiab]) AND ("school admission criteria"[MeSH] OR "admission*"[tiab] OR "student select*"[tiab]) AND english[63] |
| EMBASE | (medical education/exp/mj OR medical school*:ti,ab OR medical student*:ti,ab OR medical educat*:ti,ab) AND (interview/exp/mj OR interview*:ti,ab) AND (school admission/exp/mj OR admission*:ti,ab OR student select*:ti,ab) AND [64]/lim |
| ERIC | (DE("medical education") OR AB("medical school*") OR TI("medical school*") OR TI("medical student*") OR AB("medical student*") OR TI(“medical educat*”) OR AB("medical educat*")) AND (DE("interviews") OR AB("interview*") OR TI("interview*")) AND (DE("admission (school)") OR DE("admission criteria") OR DE("selection") OR AB("admission*") OR TI("admission*") OR AB("student select*") OR TI("student select*")) |
| CINAHL | (MH("Education, Medical+") OR AB("medical school*") OR TI("medical school*") OR AB("medical student*") OR TI("medical student*") OR AB("medical educat*") OR TI("medical educat*")) AND (MH("Interviews+") OR AB("interview*") OR TI("interview*")) AND (MH("School Admissions+") OR AB("admission*") OR TI("admission*") OR AB("student select*") OR TI("student select*")) AND LA("English") |
| PsycINFO | (DE("Medical Education") OR DE("Medical Students") OR AB("medical school*") OR TI("medical school*") OR TI("medical student*") OR AB("medical student*") OR TI(“medical educat*”) OR AB("medical educat*")) AND (DE("Interviews") OR DE("Interviewing") OR DE("Interviewers") OR AB("interview*") OR TI("interview*")) AND (AB("admission*") OR TI("admission*") OR AB("student select*") OR TI("student select*")) |
| Web of Science | (TI="medical school*" OR AB="medical school*" OR TI="medical student*" OR AB="medical student*" OR TI="medical educat*" OR AB="medical educat*") AND (TI="interview*" OR AB="interview*") AND (TI="admission*" OR AB="admission*" OR TI="student select*" OR AB="student select*") |
