# Supplementary material for: Best practices for interviewing applicants for medical school admissions: a systematic review
Source: Perspect Med Educ. 2022 Sep 22;11(5):239–46. doi: 10.1007/s40037-022-00726-8 (PMC9510545; doi:10.1007/s40037-022-00726-8)
Supplement: Supplementary file 2 — ESM 2: Characteristics of included studies and interventions [file 40037_2022_726_MOESM2_ESM.docx]

**ESM 2: Characteristics of included studies and interventions**

| **Study (Year)** | **Country** | **Study Design** | **Population** | **Intervention (Duration)** | **Control (Duration)** | **Interview Topics** |
| --- | --- | --- | --- | --- | --- | --- |
| **Shaw et al. (1995)** | USA | COS | Medical University of South Carolina applicants  (I = 245, C = 226) | 1 unstructured interview, interviewers blinded to MCAT and GPA (N/A) | 1 unstructured interview, interviewers received MCAT and GPA (N/A) | Traits of a good physician |
| **Albanese et al. (2003)** | USA | COS | University of Wisconsin Medical School applicants (I=80, C=95) | Semi-structured interviews (N/A) | Unstructured interviews (N/A) | N/A |
| **Reiter et al. (2006): Study 1** | Canada | RCT | McMaster University Faculty of Health Sciences applicants  (I = 24, C = 33) | Applicants were sent summaries of all 9 MMI stations 2 weeks prior (N/A) | Applicants were not sent summaries (N/A) | N/A |
| **Reiter et al. (2006): Study 2** | Canada | RCT | McMaster University Faculty of Health Sciences applicants  (I = 192, C = 192) | Applicants were sent a summary of 1 pilot station for a 12 station MMI (N/A) | Applicants were not sent summaries (N/A) | Personal quality domains |
| **Uijtdehaage et al. (2011)** | USA | COS | David Geffen School of Medicine at UCLA PRIME applicants  (I = 78, C = 76) | 12 station MMI and a normative rubric, difficulty increased for 1 station (N/A) | 12 station MMI and a Likert-style scoring rubric (N/A) | Speaking/argument ability and career suitability |
| **Eddins-Folensbee et al. (2012)** | USA | RCT | Baylor College of Medicine applicants (I=2345, C=1523) | 2 unstructured interviews, 1 by faculty and 1 by a student (N/A) | 2 unstructured interviews, both by faculty members (N/A) | N/A |
| **Husbands et al. (2013)** | UK | COS | Dundee Medical School applicants  (I = 140, C = 150) | MMI with 5 traditional and 5 task-based stations (70 min) | MMI with 6 traditional and 4 task-based stations (70 min) | Social skills, logic, ethics, interest, honesty, and teamwork |
| **Tiller et al. (2013)** | Australia | COS | University of Sydney international applicants  (I=293, C=571) | Virtual MMI  (63 min) | In-person MMI  (63 min) | Scenario-based questions |
| **Hissbach et al. (2014)** | Germany | COS | Hamburg Medical School  (I = 94, C = 57) | MMI with 9 stations  (45 min) | MMI with 12 stations  (60 min) | Empathy, social skills, and emotion |
| **Gay et al. (2018)** | USA | COS | University of Michigan Medical School applicants  (I = 578, C = 667) | 3 interviews, unstructured interviewers blinded to MCAT and GPA (90 min) | 3 interviews,  unstructured interviewers received MCAT and GPA (90 min) | N/A (unscripted) |
| **Kim et al. (2018)** | South Korea | RCT | Dongguk University School of Medicine applicants  (I = 16, C = 75) | MMI with 6 stations, starts with the past experience station (60 min) | MMI with 6 stations, starts with any other station (60 min) | Experience, logic, ethics, and social skills |
| **Yusoff et al. (2020)** | Malaysia | COS | Universiti Sains Malaysia School of Medical Sciences applicants  (I = 100, C = 141) | MMI with 5 stations (35 min) | Semi-structured panel interview (30 min) | Social skills, logic, ethics, interest, and language |
| **Yusoff et al. (2020)** | Malaysia | COS | Universiti Sains Malaysia School of Medical Sciences applicants  (I = 150, C = 157) | MMI with 5 stations (35 min) | Semi-structured panel interview (30 min) | Social skills, logic, ethics, interest, and language |

COS, comparative observational study; IMG, international medical graduates; min, minutes; MMI, multiple mini-interviews; N/A, not available; RCT, randomized, controlled trial; UCLA PRIME, University of California Los Angeles Program in Medical Education; UK, United Kingdom; USA, United States of America.
