# Supplementary material for: Best practices for interviewing applicants for medical school admissions: a systematic review
Source: Perspect Med Educ. 2022 Sep 22;11(5):239–46. doi: 10.1007/s40037-022-00726-8 (PMC9510545; doi:10.1007/s40037-022-00726-8)
Supplement: Supplementary file 3 — ESM 3: Intervention and measurement methods of included studies [file 40037_2022_726_MOESM3_ESM.docx]

**ESM 3: Intervention and measurement methods of included studies**

| **Study (Year)** | **Outcome** | **Assessment** | **Results** |
| --- | --- | --- | --- |
| **Shaw et al. (1995)** | Interview reliability  Interview bias (GPA, MCAT, and gender) | Interview ratings (Cronbach’s α; stepwise regression: β, R^2^) | Reliability – I: α = 0.473 vs C: 0.496  GPA – **I: β (R^2^) = 0.27 (0.07) ^a^** vs **C: 0.30 (0.16) ^a^**  MCAT – **I: 0.02 (0.02) ^a^** vs **C: 0.01 (0.02) ^a^**  Gender – **I: β = -0.12 (0.02) ^a^** vs **C: -0.20 (0.07) ^a^** |
| **Albanese et al. (2003)** | Acceptability (useful, thorough, enjoyable, better/worse than other schools, needs improvement) | Self-administered survey responses (χ^2^) | Useful – **χ^2^ = 19.27^a^**  Thorough – **42.79^a^**  Enjoyable – 1.25^b^  Better than other schools – **15.54^a^**  Worse than other schools – **18.68^a^**  Needs improvement – **35.58^a^** |
| **Reiter et al. (2006): Study 1** | Interview bias  (test disclosure) | Interview ratings (ANOVA) | I: mean (SD) = 4.97 (0.46) vs C: 4.91 (0.67)  F = 0.19^b^ |
| **Reiter et al. (2006): Study 2** | Interview bias  (test disclosure) | Interview ratings  (t-tests) | I: mean (SD) = 4.92 (1.36) vs C: 4.94 (0.65)  t = 0.24^b^ |
| **Uijtdehaage et al. (2011)** | Interview reliability  Acceptability (adequate instructions for MMI and for stations, enough time, perceived gender and cultural bias, stress) | Generalizability coefficient  Survey (7-point Likert-style questions, χ^2^) | Reliability – I: G = 0.71 vs C: G = 0.58  Enough time – I: mean (SD) = 6.4 (0.7), C: 6.4 (1.0) ^b^  Enough info for MMI – I: mean (SD) = 5.7 (0.9), C: 5.6 (0.9) ^b^  Enough info for stations – I: mean (SD) = 6.5 (0.7), C: 6.2 (1.0) ^b^  Perceived gender bias – I: mean (SD) = 6.7 (0.6), C: 6.6 (0.6) ^b^  Perceived cultural bias – I: mean (SD) = 6.6 (0.7), C: 6.3 (0.9) ^b^  Stress – **I: mean (SD) = 3.7 (1.4), C: 4.2 (1.6) ^a^** |
| **Eddins-Folensbee et al. (2012)** | Interview bias (student vs faculty interviewers) | Interview ratings (ANOVA) | 2005 – I: mean (SD) = 8.18 (1.33), C: 8.12 (1.33) ^b^  2006 – I: mean (SD) = 8.24 (1.22), C: 8.24 (1.26) ^b^  2007 – I: mean (SD) = 8.32 (1.23), C: 8.23 (1.25) ^b^  2008 – I: mean (SD) = 8.33 (1.09), C: 8.20 (1.13) ^b^  2009 – I: mean (SD) = 8.33 (1.19), C: 8.19 (1.25) ^b^  2010 – I: mean (SD) = 8.12 (1.35), C: 8.13 (1.21) ^b^ |
| **Husbands et al. (2013)** | Interview reliability  Predictive validity (exams and objective structured clinical examination [OSCE]) | Interview ratings (Cronbach’s α; Pearson’s r correlation) | Reliability – I: α = 0.69 vs C: 0.70  Year 1, Semester 1 Exams – I: r = -0.01^b^ vs C: r = 0.18^b^  Year 1, Semester 1 OSCE – I: r = -0.07^b^ vs **C: r = 0.24^a^**  Year 1, Semester 2 Exams – I: r = 0.03^b^ vs **C: r = 0.33^a^**  Year 1, Semester 2 OSCE – **I: r = 0.50^a^** vs **C: r = 0.43^a^** |
| **Tiller et al. (2013)** | Interview reliability  Interview bias  Feasibility (costs) | Interview ratings, financial costs | Reliability – I: G = 0.76, C: 0.70  Bias (virtual) – I: mean (SD) = 125.6 (1.62) vs C: 126.7 (17.2)^b^  Costs – I: $10,145, C: $61,887 |
| **Hissbach et al. (2014)** | Interview reliability  Interview bias  Feasibility (costs) | Interview ratings (ICC, statistics), financial costs | Reliability – I: ICC = 0.76 vs C: 0.75  Bias – I: mean (SD) = 3.23 (1.05) vs 3.23 (0.83)  Costs per applicant – I: $495, C: $915 |
| **Gay et al. (2018)** | Interview bias (gender, age, grade point average [GPA], MCAT) | Interview ratings (stepwise regression: β) | Gender – **I: β =0.24^a^** vs **C: 0.23^a^**  Age – **I: β =0.07^a^** vs **C: 0.07^a^**  GPA – **I: β =0.85^a^** vs **C: 0.44^a^; comparison: β = 0.573^a^**  MCAT – **I: β =0.05^a^** vs **C: 0.57^a^**; comparison: β = 0.010^b^ |
| **Kim et al. (2018)** | Interview bias (station order)  Acceptability (difficulty, anxiety) | Interview ratings (ANOVA), self-administered survey responses (ANOVA) | Bias – F = 1.12^b^  Perceived difficulty – F = 0.31 ^b^  Anxiety – F = 1.15^b^ |
| **Yusoff et al. (2020)** | Acceptability (perceived educational environment)  Convergent validity (personality, emotional intelligence, and stressors) | Self-administered survey responses (t-tests): Universiti Sains Malaysia Emotional Quotient Inventory, Universiti Sains Malaysia Personality Inventory, Dundee Ready Educational Environment Measure, Medical Student Stressor Questionnaire | Social competence – I: mean (SD) = 3.0 (0.7) vs C: 2.9 (0.8)  Personal competence – I: 2.9 (0.5) vs C: 2.9 (0.6)  Emotional intelligence – I: 2.9 (0.5) vs C: 2.9 (0.7)  Extraversion – I: 8.0 (2.4) vs C: 8.5 (2.5)  **Conscientiousness – I: 7.6 (2.1) vs C: 8.2 (2.1)**  Agreeableness – I: 8.4 (2.3) vs C: 8.8 (2.2)  Neuroticism – I: 4.2 (2.2) vs C: 3.9 (2.4)  Openness – I: 9.0 (2.2) vs C: 8.9 (2.2)  Perception of learning – I: = 6.4 (1.8) vs C: 6.1 (1.8)  Perception of teaching – I: 6.2 (1.9) vs C: 6.1 (2.0)  Academic self-perception – I: 6.1 (1.5) vs C: 6.0 (1.9)  Perception of atmosphere – I: 10.6 (2.6) vs C: 9.9 (2.8)  **Social self-perception – I: 6.7 (2.0) vs C: 6.0 (2.0)**  Academic stress – I: 2.8 (0.7) vs C: 2.8 (0.8)  Group stress – I: 2.2 (0.8) vs C: 2.2 (0.9)  Social stress – I: 2.3 (0.8) vs C: 2.2 (0.9)  Interpersonal stress – I: 2.2 (0.9) vs C: 2.0 (1.1)  Drive stress – I: 1.4 (1.1) vs C: 1.3 (1.1)  Teaching/learning stress – I: 1.8 (0.9) vs C: 1.8 (1.0) |
| **Yusoff et al. (2020)** | Predictive validity (stress, anxiety, depression, burnout) | Self-administered survey responses (t-tests): Depression Anxiety Stress Scale, CBI | Stress – I: mean (SD) = 16.8 (8.7) vs C: 15.7 (10.5)  Anxiety – I: 16.3 (9.0) vs C: 15.3 (9.6)  Depression – I: 11.4 (9.5) vs C: 10.6 (9.8)  Personal burnout – I: 12.3 (4.8) vs 12.1 (5.1)  Work burnout – 12.8 (4.6) vs 12.5 (5.2)  Client burnout – 9.1 (4.8) vs 8.5 (5.0) |

ANOVA, Analysis of Variance; CBI, Copenhagen Burnout Inventory; ICC, intraclass correlation; MCAT, Medical College Admission Test; SD, standard deviation. Statistically significant correlations are in bold.
